# Contracted thalamic shape is associated with early development of levodopa-induced dyskinesia in Parkinson’s disease

**DOI:** 10.1038/s41598-022-16747-6

**Published:** 2022-07-25

**Authors:** Han Soo Yoo, Eun-Chong Lee, Seok Jong Chung, Byoung Seok Ye, Young H. Sohn, Joon-Kyung Seong, Phil Hyu Lee

**Affiliations:** 1 Department of Neurology, Gangnam Severance Hospital, Yonsei University College of Medicine, Seoul, South Korea; 2 School of Biomedical Engineering, Korea University, 145, Anam-ro, Seongbuk-gu, Seoul, 02841 South Korea; 3 Department of Neurology, Yongin Severance Hospital, Yonsei University College of Medicine, Yongin, South Korea; 4 Department of Neurology, Yonsei University College of Medicine, 50 Yonsei-ro, Seodaemun-gu, Seoul, 03722 South Korea; 5 Department of Artificial Intelligence, Korea University, Seoul, South Korea; 6 Interdisciplinary Program in Precision Public Health, Korea University, Seoul, South Korea; 7 Severance Biomedical Science Institute, Yonsei University College of Medicine, Seoul, South Korea

**Keywords:** Neuroscience, Neurology

## Abstract

Levodopa-induced dyskinesia (LID), a long-term motor complication in Parkinson’s disease (PD), is attributable to both presynaptic and postsynaptic mechanisms. However, no studies have evaluated the baseline structural changes associated with LID at a subcortical level in PD. A total of 116 right-handed PD patients were recruited and based on the LID latency of 5 years, we classified patients into those vulnerable to LID (PD-vLID, n = 49) and those resistant to LID (PD-rLID, n = 67). After adjusting for covariates including dopamine transporter (DAT) availability of the posterior putamen, we compared the subcortical shape between the groups and investigated its association with the onset of LID. The PD-vLID group had lower DAT availability in the posterior putamen, higher parkinsonian motor deficits, and faster increment in levodopa equivalent dose than the PD-rLID group. The PD-vLID group had significant inward deformation in the right thalamus compared to the PD-rLID group. Inward deformation in the thalamus was associated with an earlier onset of LID at baseline. This study suggests that independent of presynaptic dopamine depletion, the thalamus is a major neural substrate for LID and that a contracted thalamic shape at baseline is closely associated with an early development of LID.

## Introduction

Despite the undoubted usefulness of levodopa in the treatment of Parkinson’s disease (PD), prolonged treatment with levodopa can be complicated by levodopa-induced dyskinesia (LID), which causes disabling involuntary movements. LID usually presents with chorea/choreoathetoid movements, which can be troublesome and require intervention. It is also associated with a poor quality of life and high health-related costs^1^. The reported incidence rate of LID is approximately 30–80%^2^. As such, LID, a long-term motor complication, poses a major challenge to the pharmacologic treatment of PD.

Both presynaptic and postsynaptic mechanisms are known to contribute to the development of LID. Nigral dopamine neuron loss and pulsatile stimulation of dopamine receptors interfere with the maintenance of stable and physiologic synaptic and extra-synaptic dopamine levels^3^. It leads to pre- and postsynaptic plastic changes in the striatum, cortex, and its connections^4^. Presynaptically, a dopamine transporter (DAT) positron emission tomography (PET) study showed that striatal dopamine depletion determines the timing of LID^5^. Postsynaptically, a resting-state functional magnetic resonance imaging (MRI) study revealed that dopaminergic modulation of resting-state connectivity between the putamen and primary sensorimotor cortex predicted the development of LID^6^. Besides, a task-specific functional MRI study provided evidence of overactivity in the supplementary motor area and underactivity in the right inferior prefrontal gyrus in PD patients with LID^7^. Despite many functional studies investigating the neural substrates of LID, no studies have evaluated the structural changes associated with LID at a subcortical level.

Subcortical deep nuclei, especially the basal ganglia and thalamus, are essential components for appropriate motor control in PD. As parts of cortico-basal ganglia-thalamo-cortical motor circuit, the basal ganglia are associated with the control of voluntary motor movement and motor learning^8^ and the thalamus participates in processing and relaying information to the motor cortices^9^. Furthermore, these structures are targets for deep brain stimulation in PD, leading to alleviation of motor deficits and LID^10^. Therefore, structural or functional change in subcortical deep nuclei at postsynaptic level may play a crucial role in motor phenomena in PD, and should be considered important neural substrates for the pathophysiology of LID.

Here, we hypothesized that in drug-naïve patients with PD, deformities in the subcortical structures might contribute to the development of LID later in the disease course, independent of the presynaptic dopamine depletion. We used a shape analysis for subcortical structures since it has been reported that a surface feature at the vertex level is more sensitive to reflect changes in the subcortical structures than a volume in PD^11,12^. Thus, we explored the shape deformation of subcortical structures in patients with PD according to the vulnerability to LID using a surface-based shape analysis procedure to elucidate the role of subcortical shape in the pathophysiology of LID.

## Results

### Demographic characteristics of the study subjects

The baseline demographic characteristics of the patients are summarized in Table 1. The PD-vLID group showed a higher UPDRS part III score (*P* < 0.001) and lower DAT availability in the posterior putamen (*P* = 0.048) than did the PD-rLID group at the time of diagnosis. There were no differences in age, sex, interval between PD onset to MRI scanning, PD duration, follow-up duration, duration of levodopa therapy, BMI, total K-MMSE score, onset side, intracranial volume, and vascular risk factors (hypertension, diabetes mellitus, dyslipidemia, ischemic heart disease, or ischemic stroke) between the PD-vLID and PD-rLID groups. Of the 116 patients enrolled, 83 patients developed LID during the follow-up period, and the mean time from PD onset to the development of LID was 4.2 ± 1.9 years. As expected, LID increments per year were faster in the PD-vLID group than in the PD-rLID group (P = 0.007).

**Table 1:** Demographics and clinical characteristics of patients with Parkinson’s disease.

Variables	PD-vLID (n = 49)	PD-rLID (n = 67)	*P*-value
Age at onset, y	63.8 ± 9.4	65.2 ± 8.3	0.344
Sex, female, n (%)	30 (61.2)	36 (53.7)	0.689
Onset to MRI scanning, y	1.5 ± 1.4	1.2 ± 0.9	0.105
PD duration, y	8.9 ± 2.4	9.4 ± 1.5	0.186
Follow-up duration, y	7.3 ± 2.0	8.1 ± 1.4	0.114
Duration of levodopa therapy, y	7.0 ± 1.3	7.9 ± 1.3	0.135
Body mass index, kg/m^2^	23.0 ± 3.3	23.5 ± 2.8	0.566
MMSE	26.5 ± 2.5	26.8 ± 2.6	0.085
UPDRS part III score	26.6 ± 11.6	17.5 ± 10.1	< 0.001
**Onset side, n (%)**			0.520
Dominant side	24 (49.0)	30 (44.8)	
Non-dominant side	19 (38.8)	32 (47.8)	
Equivocal	6 (12.2)	5 (7.5)	
LED increment per year, mg/y	202.4 ± 133.3	109.5 ± 37.1	0.007
Intracranial volume, mm^3^	1263.0 ± 198.9	1317.0 ± 240.7	0.160
DAT availability in the posterior putamen	1.1 ± 0.4	1.4 ± 0.5	0.048
**Vascular risk factors**			
Hypertension	17 (34.7)	25 (37.3)	0.563
Diabetes mellitus	3 (6.1)	11 (16.4)	0.122
Dyslipidemia	11 (22.4)	7 (10.4)	0.133
Ischemic heart disease	4 (8.2)	8 (11.9)	0.509
Ischemic stroke	2 (4.1)	1 (1.5)	0.295

Values are expressed as the mean ± standard deviation or number (percentage) as appropriate.

DAT, dopamine transporter; LED, levodopa-equivalent dose; MMSE, Mini-Mental State Examination; MRI, magnetic resonance imaging; PD, Parkinson’s disease; UPDRS, Unified PD Rating Scale.

### Comparison of the subcortical shape between the PD-rLID and PD-vLID groups

**Figure 1:**
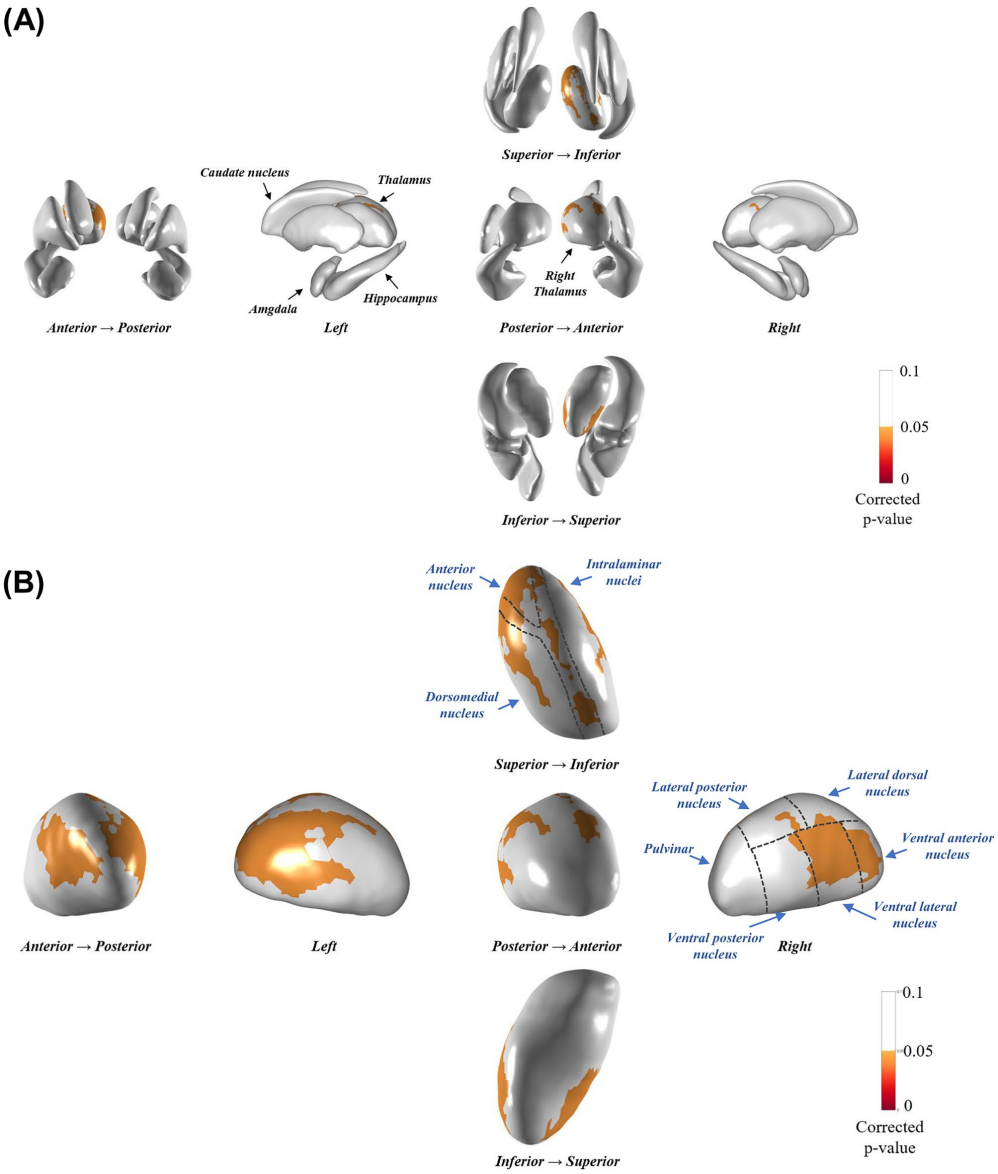
Comparison of the regional subcortical shape between the PD-rLID and PD-vLID groups. The results are based on an analysis of covariance, after adjusting for age, sex, disease duration, levodopa-equivalent dose increments per year, dopamine transporter availability in the posterior putamen, and intracranial volume. The cluster-based statistics-corrected statistical map (*P*-map) indicates the subcortical regions that showed significant inward deformation in the latter group at the vertex level (yellow to red color). PD-rLID, PD group resistant to levodopa-induced dyskinesia; PD-vLID, PD group resistant to levodopa-induced dyskinesia.

We performed a comparative analysis of the subcortical shape between the PD-rLID and PD-vLID groups after controlling for age at onset, sex, disease duration, LED increments per year, DAT availability in the posterior putamen, and intracranial volume. The PD-vLID group had more inward deformation in the right thalamus with cluster size 813 (Fig. 1A), especially in the ventral anterior, ventral lateral, intralaminar, anterior, and mediodorsal nuclei (Fig. 1B), compared to the PD-rLID group. The mean subcortical shape values were also compared between the two groups (Supplementary Table S1). Among the 12 regions of the bilateral subcortical structures, the PD-vLID group had smaller mean subcortical shape values in the left, right, and bilateral thalami than the PD-rLID group. The mapping of *P*-values between the groups obtained from F-statistics before multiple corrections of cluster size is provided in Supplementary Fig. S1.

### Association of the subcortical structures with motor and cognitive function at baseline and onset of LID

**Figure 2:**
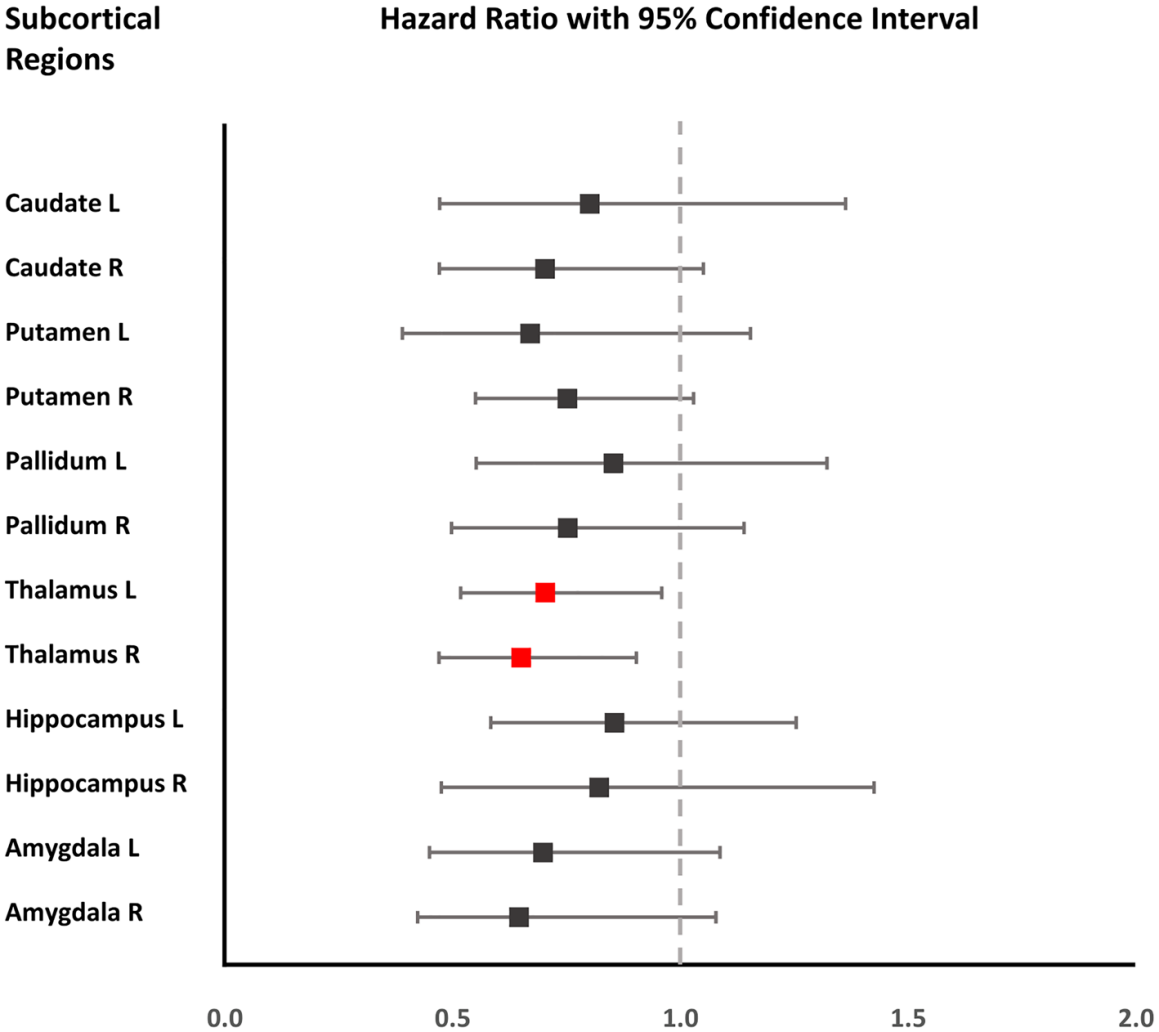
Association between the subcortical structures and onset of LID. A forest plot showing the hazard ratio and 95% confidence intervals of the early onset of LID according to the subcortical structures. The results are based on a Cox proportional hazard model, after adjusting for age, sex, disease duration, levodopa-equivalent dose increments per year, dopamine transporter availability in the posterior putamen, and intracranial volume. The red square indicates the subcortical structures that are significantly associated with the onset of LID.

In partial correlation analysis using the same covariates, mean putaminal shape value was negatively associated with UPDRS part III score (correlation coefficient − 0.260; *P* = 0.006), while other subcortical shape values were not. None of mean subcortical shape values were associated with total K MMSE score (Supplementary Table S2). The Cox proportional hazard model revealed that patients with PD who had inward deformation in the right or left thalamus from an early stage had a higher risk of developing early-onset LID after controlling for the covariates, including DAT availability in the posterior putamen (left thalamus, HR 0.70, 95% CI 0.51–0.95; *P* = 0.023; right thalamus, HR 0.64, 95% CI 0.46–0.90, *P* = 0.009; Fig. 2 and Supplementary Table S3). In addition, patients with PD who had inward deformation in the right caudate or putamen tended to have a higher risk of LID development (right caudate, HR 0.70, 95% CI 0.46–1.04; *P* = 0.079; right putamen, HR 0.75, 95% CI 0.54–1.02, *P* = 0.068).

## Discussion

The present study analyzed the association between baseline subcortical shape deformation and the development of LID in drug-naïve patients with PD. The major findings were as follows. First, the mean latency of LID after levodopa intake was 4.2 years. Furthermore, patients with PD who were vulnerable to LID development had lower DAT availability in the posterior putamen and higher parkinsonian motor deficits than those resistant to LID. Second, patients with PD who were vulnerable to LID had significant inward deformation in the right thalamus, especially the ventral anterior, ventral lateral, intralaminar, anterior, and mediodorsal nuclei, compared to those resistant to LID, independent of presynaptic dopamine depletion. Third, inward deformation in the putamen at baseline was associated with baseline motor dysfunction, while inward deformation in the thalamus at baseline was associated with an earlier onset of LID. Taken together, these findings suggest that independent of the presynaptic dopamine depletion, the thalamus may be a major neural substrate for the development of LID.

Although the thalamus is generally believed to be a relay station of multiple sensory information, it also plays an important role in motor control^13^. The thalamus acts as a modulator or integrator between motor areas of the cerebral cortex and motor-related subcortical structures, such as the cerebellum and basal ganglia^9^. In the basal ganglia circuit, the thalamus receives inhibitory input from internal globus pallidus and substantia nigra pars reticulata and gives an excitatory signal to the motor-related cortex. Therefore, we can infer that the thalamus may undergo a downstream change as an adaptation to postsynaptic plasticity and play a crucial role in the development of LID. The postsynaptic changes underlying LID have been reported as changes in the gene and protein expression in striatal dopamine receptors^14^, dissociation between blood flow and metabolism in the striatum, and functional and structural changes in the motor-related cortex^6,7^. Here, we first investigated subcortical structural changes associated with motor deficits at baseline and the development of LID. We found that local thalamic inward deformation at an early stage of PD was not associated with baseline motor or cognitive dysfunction but with earlier onset of LID later. Considering functional connectivity in the cortico-basal ganglia-thalamo-cortical circuit in PD with LID, hypersensitive dopamine receptors result in hypoactivity of the internal globus pallidus. This leads to disinhibition of the thalamocortical projection, facilitating the abnormal recruitment of cortical motor areas, ultimately giving rise to dyskinetic movements^15^. Thus, we can infer from our results that inward deformation in thalamic shape may not be directly associated with parkinsonian motor symptom but with long-term motor complication, probably due to vulnerability to postsynaptic plasticity. Further studies are necessary to investigate the relationship between postsynaptic striatal plastic change and thalamic response.

Patients with PD who were vulnerable to LID showed inward deformation in the ventral anterior, ventral lateral, mediodorsal, intralaminar, and anterior nuclei. The ventral anterior and ventral lateral parts are generally termed the motor thalamus, which comprises the basal ganglia motor circuit^16^. A parkinsonian primate study revealed that metabolic activity in these areas was significantly decreased in dyskinetic monkeys^17^. Given our results, dysfunction of the motor thalamus accompanied by locally atrophied motor thalamus may be a major risk factor for dyskinetic presentation in patients with PD. The centromedian-parafascicular nuclei complex (CM/PF), a part of the intralaminar nucleus of the thalamus, is a major source of direct glutamatergic input to the striatum and connects to the fronto-parietal cortex and other subcortical structures^18^. The CM/Pf complex has been recently found to exhibit marked neuronal loss both in PD and in parkinsonian animal models based on neurotoxic DA lesions^19^. Thalamic deep brain stimulation in the CM/Pf complex was shown to be related to the improvement of LID^20^. Thalamostriatal input from the CM/Pf complex may also play an important role in plastic adaptation in this system and PD motor behavior^21^. In line with our study, these studies suggest an important role of the CM/Pf complex in LID.

In addition, the mediodorsal nucleus of the thalamus has connectivity with the prefrontal cortex and has been reported to be involved in regulating flexibility of prefrontal-dependent cognitive function^22^. The mediodorsal nucleus, as one of higher-order nuclei of the thalamus, modulates cortico-cortical communication^23^, and its responsivity was modulated by dopamine^24^. A link between LID and frontal cognitive dysfunction has also been reported in PD^25^. Together with the findings in our study, we can infer that atrophy in the mediodorsal nucleus is closely associated with prefrontal dysfunction and long-term pulsatile stimulation of dopamine receptor may affect mediodorsal nucleus-mediated prefrontal cortical plasticity, which may lead to maladaptive responsiveness to dopamine administration presenting as LID. The exact pathophysiological role of the mediodorsal nucleus and its connection with the prefrontal cortex in LID should be investigated in future.

Thalamotomy or thalamic deep brain stimulation was previously attempted to alleviate LID in PD^20,26^, although it was soon replaced by other targets, particularly the subthalamic nucleus and internal globus pallidus. Some studies have shown improvement of LID when the ventral oralis complex^26^, CM/PF^20^, or thalamic areas that receive efferents from pallidum^27^ were targeted, showing that the thalamus contributes to the development of LID. Here, we also demonstrated that the thalamic shape before taking levodopa could determine the occurrence of LID, regardless of postsynaptic plastic changes during long-term levodopa intake. This suggests that the normal thalamic shape and function may withstand long-standing electrophysiologic or molecular changes in the basal ganglia associated with LID and convey normal excitatory projection to the cortex.

Our subcortical mapping showed a significant shape difference in the right side, i.e., non-dominant hemisphere, of the thalamus according to the vulnerability to LID. The level of dopamine tended to be higher in the basal ganglia of the dominant hemisphere^28^, and cortical atrophy in motor-related cortical areas was only observed in PD patients with non-dominant side onset^29^. Significant shape differences were detected between the left and right thalami in PD^30^. These studies suggest that there may be a difference in the cortico-basal ganglia-thalamo-cortical motor circuit between the dominant and non-dominant hemispheres in patients with PD. Our previous studies showed that right-handed PD patients with dominant side onset of parkinsonism showed fewer motor deficits^31^, and that LID developed earlier in the non-dominant side than in the dominant side in patients with PD, suggesting a greater neural reserve or potential of synaptic plasticity in the dominant hemisphere^32^. Given the pathophysiology of LID, it can be inferred that patients with thalamic abnormalities in the non-dominant hemisphere have poorer adaptation to aberrant plasticity.

This study had several limitations. First, a 5-year time window to classify patients into LID-vulnerable and LID-resistant groups may be arbitrary, although the cutoff year was based on a previous study^33^. It is necessary to find a better biomarker that can reflect the actual vulnerability to LID to elucidate neural substrates for LID. Second, the onset date of LID was determined by the retrospective recall of the patients or their caregivers or by neurological examination during the outpatient clinic. Thus, the onset of these complications might have occurred earlier than reported. Third, we did not measure the severity of LID, which could give us additional information on the association between changes in the subcortical structures and LID. Fourth, a substantial number of patients were excluded at baseline and after longitudinal evaluation, which might have biased the results. However, the strict application of the inclusion and exclusion criteria, while adjusting for appropriate covariates, helped to more clearly show the independent relationship between LID and the subcortical shape.

In conclusion, the present study suggests that regardless of the presynaptic dopamine depletion as well as other possible risk factors for LID, early inward deformation in the thalamus could be independently associated with the likelihood of developing early LID with long-term levodopa treatment. These results provide a new perspective in the investigation of the role of the thalamus in PD and its contribution to the pathophysiology of LID.

## Methods

### Subjects

In this retrospective cohort study, a cohort of patients with drug-naïve PD who had visited the movement disorders outpatient clinic at Severance Hospital, Yonsei University Health System, between January 2010 and December 2014, were involved. Patients underwent brain MRI and *N*-(3-[^18^F]fluoropropyl)-2β-carbomethoxy-3β-(4-iodophenyl) nortropane (^18^F-FP-CIT) PET on the same day within a month after their first visit. Parkinsonian motor severity was assessed at the time of ^18^F-FP-CIT PET acquisition using the Unified PD Rating Scale-part III (UPDRS-III). Then, the patients started dopamine replacement therapy based on clinical history, neurological examination, and MRI and PET scan findings.

**Figure 3:**
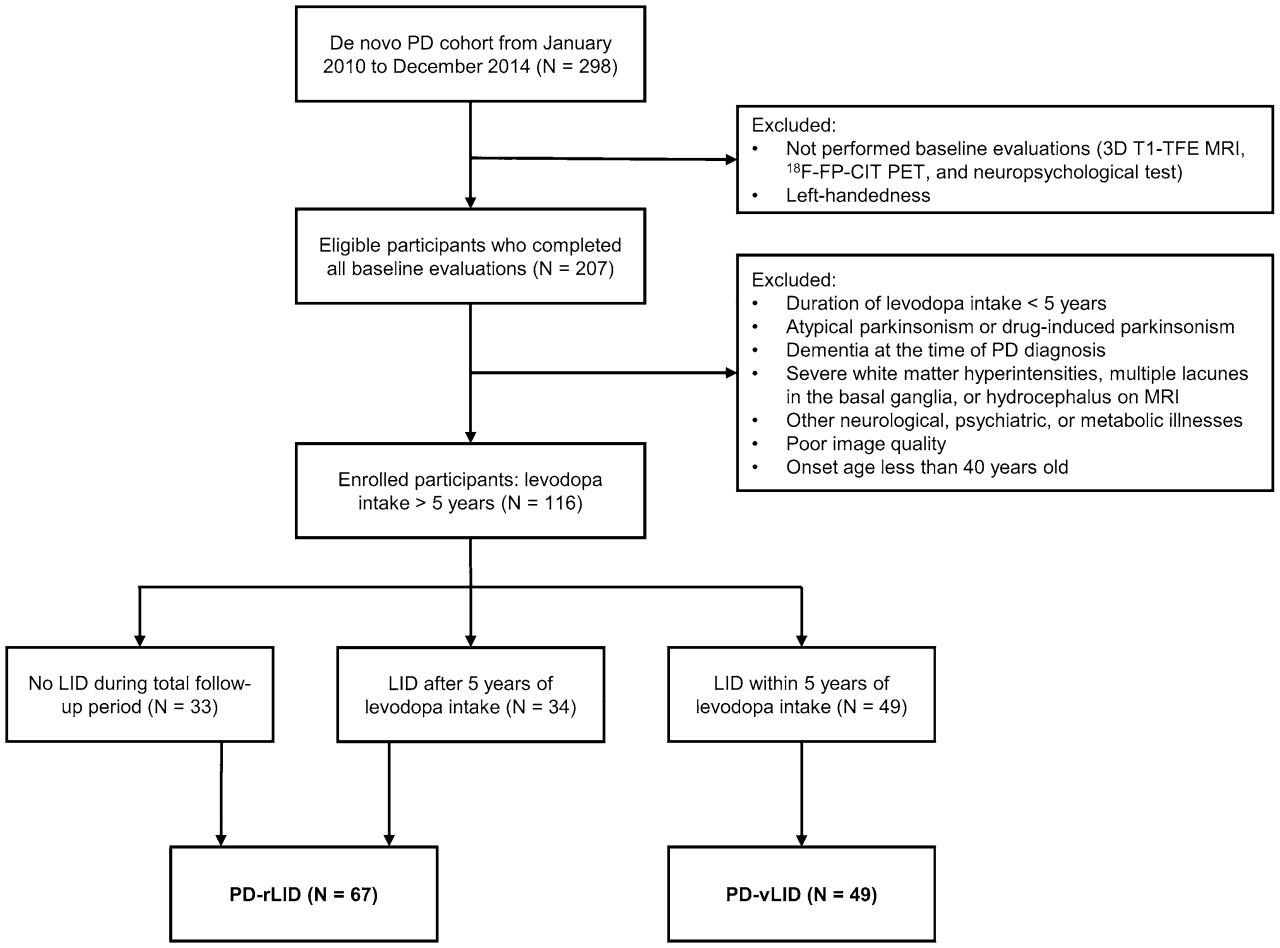
Flowchart of enrollment of the study subjects.

Among the patients in the cohort, we enrolled a total of 116 patients who met the following inclusion criteria: (1) had a diagnosis of PD based on the clinical diagnostic criteria of the United Kingdom PD Society Brain Bank^34^; (2) underwent a three-dimensional volumetric brain MRI and ^18^F-FP-CIT PET at baseline; (3) had decreased DAT availability in the posterior putamen, which was interpreted by a nuclear medicine physician blinded to the clinical status of the patients; (4) had been taking levodopa for more than 5 years; and (5) had documented right-handedness. The exclusion criteria were as follows: (1) atypical parkinsonism or drug-induced parkinsonism; (2) dementia at the time of baseline evaluation^35^; (3) severe white matter hyperintensities, multiple lacunes in the basal ganglia, or hydrocephalus on MRI; (4) other neurologic, psychiatric, or metabolic illnesses; (5) poor image quality; and (6) illness onset age of less than 40 years. Details of the enrolled study subjects are illustrated in Fig. 3.

The onset side of parkinsonism was defined based on a previous study^32^. The levodopa-equivalent dose (LED) was calculated according to a previously described method^36^. Increments in the LED per year were calculated as follows: (total LED at the last visit − total LED at the first prescription)/total duration of levodopa treatment. Baseline height and weight were measured at the initial visit, and body mass index (BMI) was calculated by dividing weight expressed in kilograms by the square product of height expressed in meters. The Korean version of the Mini-Mental State Examination (K-MMSE) was used to assess general cognition.

The patients generally visited our outpatient clinic every 3 to 6 months, and two movement disorders experts (Y.H.S. and P.H.L.) carefully assessed the presence of LID through patient history and details obtained from caregivers or by direct neurologic examination at every visit. We regarded the date on which the patients with PD or their caregivers reported that the LID occurred or the date on which LID was first seen in the clinic as the date of LID occurrence. Since the cumulative incidence of LID increases with the duration of levodopa intake and the mean latency from the initiation of levodopa treatment to LID in patients with PD was approximately 5 years^33^, we divided the PD patients into two groups: a LID-resistant group (PD-rLID, n = 67) who had developed LID after 5 years of levodopa treatment or had never developed LID during more than 5 years of follow-up and a LID-vulnerable group (PD-vLID, n = 49) who had developed LID within 5 years of levodopa administration.

This study was approved by our institutional review board of the Yonsei University Medical Center. All participants provided written informed consent. All research was performed in accordance with relevant guidelines and regulations.

### MRI acquisition

All MRI scans were acquired using a Philips 3.0-T scanner (Philips Intera; Philips Medical System, Best, The Netherlands) with a SENSE head coil (SENSE factor = 2). A high-resolution T1-weighted MRI volume data set was obtained from all subjects using a three-dimensional T1-TFE sequence configured with the following acquisition parameters: axial acquisition with a 224 × 256 matrix; 256 × 256 reconstructed matrix with 182 slices; 220 mm field of view; 0.98 × 0.98 × 1.2 mm3 voxels; TE (echo time), 4.6 ms; TR (repetition time), 9.6 ms; flip angle, 8°; and slice gap, 0 mm.

### ^18^F FP-CIT PET image acquisition and quantitation

^18^F-FP-CIT PET scan was obtained using a Discovery 600 system (General Electric Healthcare, Milwaukee, MI, USA). A dose of 185 MBq (5 mCi) of FP-CIT was injected intravenously during the procedure. Ninety minutes after injection, images were acquired over a 20-min session following CT scanning for attenuation correction. Spiral CT scanning was performed with a 0.8 s/rotation at 120 kVp, 10 mA, 3.75 mm slice thickness, 0.625 mm collimation and 9.375 mm table feed per rotation. Images were reconstructed using the ordered subset expectation maximization (OSEM) algorithm with 4 iterations and 32 subsets. Gaussian filter with 4 mm full-width at half-maximum (FWHM) was applied to reconstructed PET images, which were 256 × 256 matrices with 0.98 mm pixel and 0.98 mm slice thickness.

Image processing was performed using MATLAB (The MathWorks, Inc, Natick, MA, USA) software for statistical parametric mapping 8 (SPM8) and ITK-SNAP (http://www.itksnap.org). All reconstructed ^18^F-FP-CIT images were normalized to the ^18^F-FP-CIT template, which was constructed using the ^18^F-FP-CIT PET images and T1-weighted MR images of 40 healthy controls, as described previously^37^. All healthy controls from which the ^18^F-FP-CIT template was derived, had no previous history of neurologic or psychiatric illness. They showed normal cognitive function in all neuropsychological tests, and exhibited normal findings on neurologic examination, structural MRI, and ^18^F-FP-CIT PET. Twelve volumes-of-interest (VOI) of striatal subregions and one occipital VOI were drawn on the ^18^F-FP-CIT template, as described previously^38^. In brief, using the anterior–posterior commissure line on the transverse plane, the striatum was divided into dorsal and ventral portions. The ventral portion comprised two subregions; the ventral striatum and the ventral putamen. The dorsal portion was divided into anterior and posterior subregions along the coronal anterior commissure plane: anterior caudate, posterior caudate, anterior putamen, and posterior putamen. The level of DAT availability in each VOI was calculated in terms of the specific to non-specific binding ratio as follows: (mean standardized uptake of striatal subregional VOI – mean standardized uptake of occipital VOI)/mean SUV of occipital VOI. Occipital uptake was regarded as non-specific binding.

### Shape analysis of the subcortical structures

To measure the shape deformation of the subcortical structures, the surface mesh for each structure was constructed using the volumetric data extracted through the FreeSurfer software package (version 6.0.0; Athinoula A. Martinos Center at the Massachusetts General Hospital, Harvard Medical School; http://www.surfer.nmr.mgh.harvard.edu/). Surface meshes of segmented subcortical nuclei represent the volumetric information of each nucleus and are composed of parameterized deformable surfaces. According to the methodology used in a previous paper, the native surface was created through the Laplacian-based surface deformation process of the template surface^39–41^. The number of vertices of the template surface was 2562 and that in each subcortical surface was unified to 2562. Then, the surface registration method developed by Cho et al*.* was used to establish the vertex correspondence of subcortical surface mesh across the sample^42^. After projecting the vertex locations from each nucleus onto the surface of each subcortical mesh, the approximated local shape volume for each vertex was calculated by employing the method proposed by Shapira et al*.*^43^. After shooting a ray that heads inward from the surface to the volume, the distance until it hits the opposite surface is defined as the inward distance. The weighted sum of the inward distances was calculated to compare how much shrinkage occurred relative to the surrounding surface. By definition, this shape value represents the local volume at each vertex. Therefore, a decrease in the local volume was considered to indicate inward deformation in that area. For noise removal, the discrete Laplacian matrix was calculated for each subcortical surface mesh, and the local shape values with high frequency were eliminated as noise. Also, of the regions statistically significant in the subcortical structure, clusters with a small number of vertices were considered noise and removed.

### Statistical analysis

Baseline demographic characteristics of the study subjects were analyzed using an independent *t*-test for continuous variables and Pearson’s *χ*^2^-test or Fisher’s exact test for categorical variables as appropriate. For the group-wise comparisons of vertex-wise subcortical shape, an analysis of covariance was performed based on the methodology introduced in a previous paper^44^. Age, sex, disease duration, LED increments per year, and intracranial volume were used as covariates. Furthermore, we also included DAT availability of the posterior putamen as one of the covariates to adjust the difference in DAT availability of the posterior putamen between the two groups and to exclude the contribution of presynaptic dopamine depletion to the development of LID. UPDRS part III score was not included as a covariate in the analysis of covariance because disease duration and DAT availability in the posterior putamen were significantly correlated with UPDRS part III score (for disease duration, correlation coefficient 0.330, *P* < 0.001; for DAT availability in the posterior putamen, correlation coefficient − 0.259, *P* = 0.005). F-statistics was calculated through a group comparison with 5000 permutations for each vertex. The cluster size of vertices with an F-statistic value larger than 0.3 was compared with the cluster sizes in each permutation, and then the *P*-value was calculated for each cluster. In this way, the multiple corrections for the *P*-value of each structure were done using cluster-based statistics in the subcortical shape analyses. Group-wise comparison of mean subcortical shape value was also performed using the analysis of covariance with the same covariates as in the analysis of vertex-wise subcortical shape. Partial correlation analyses were used to examine the association of mean subcortical shape value with baseline motor and cognitive function using the same covariates. The hazard ratio (HR) and 95% confidence intervals (CIs) of the early onset of LID according to the subcortical structures were calculated using a Cox proportional hazards model that included the same covariates. The data were analyzed with SPSS software version 23 (IBM Corp, Armonk, NY). *P-*values of < 0.05 were considered statistically significant.

## Supplementary Information

Supplementary Information.

## Data Availability

The datasets generated and/or analyzed during the current study available from the corresponding author on reasonable request.
