# Supplementary material for: Contracted thalamic shape is associated with early development of levodopa-induced dyskinesia in Parkinson’s disease
Source: Sci Rep. 2022 Jul 25;12:12631. doi: 10.1038/s41598-022-16747-6 (PMC9314442; doi:10.1038/s41598-022-16747-6)
Supplement: Supplementary file 1 — Supplementary Information. [file 41598_2022_16747_MOESM1_ESM.doc]

**Supplementary Materials**

**Supplementary Tables**

**S1.** Group-wise comparison of mean subcortical shape value

**S2.** Association between mean subcortical shape value and baseline motor and cognitive function

**S3.** Cox regression analysis of subcortical shape for the development of levodopa-induced dyskinesia

**Supplementary Figure**

**S1.** Uncorrected *P*-map of the regional subcortical shape between the PD-rLID and PD-vLID

**Supplementary Table S1.** Group-wise comparison of mean subcortical shape value

| Subcortical region | Side | PD-vLID | PD-rLID | *P* value |
| --- | --- | --- | --- | --- |
| Caudate | Left | 5.00 (0.08) | 5.07 (0.06) | 0.483 |
|  | Right | 5.03 (0.10) | 5.24 (0.08) | 0.106 |
|  | Bilateral | 5.02 (0.08) | 5.15 (0.07) | 0.191 |
| Putamen | Left | 8.44 (0.08) | 8.58 (0.07) | 0.213 |
|  | Right | 7.89 (0.10) | 8.13 (0.09) | 0.107 |
|  | Bilateral | 8.17 (0.09) | 8.35 (0.07) | 0.117 |
| Pallidum | Left | 7.46 (0.10) | 7.45 (0.08) | 0.892 |
|  | Right | 6.50 (0.10) | 6.49 (0.08) | 0.489 |
|  | Bilateral | 6.93 (0.09) | 6.97 (0.08) | 0.758 |
| Thalamus | Left | 13.68 (0.14) | 14.04 (0.12) | 0.048 |
|  | Right | 14.20 (0.11) | 14.61 (0.10) | 0.008 |
|  | Bilateral | 13.94 (0.12) | 14.32 (0.10) | 0.014 |
| Hippocampus | Left | 6.99 (0.10) | 7.12 (0.09) | 0.363 |
|  | Right | 6.91 (0.08) | 6.98 (0.07) | 0.556 |
|  | Bilateral | 6.95 (0.08) | 7.04 (0.07) | 0.370 |
| Amygdala | Left | 5.79 (0.09) | 5.91 (0.08) | 0.284 |
|  | Right | 5.88 (0.09) | 6.03 (0.07) | 0.198 |
|  | Bilateral | 5.84 (0.08) | 5.96 (0.06) | 0.142 |

Values are presented as estimated mean (standard error). Data are results of analysis of covariance, adjusting for age, sex, disease duration, levodopa-equivalent dose increment per year, dopamine transporter availability in the posterior putamen, and intracranial volume.

**Supplementary Table S2. Association between mean subcortical shape value and baseline motor and cognitive function**

|  | **Caudate** | **Putamen** | **Pallidum** | **Thalamus** | **Hippocampus** | **Amygdala** |
| --- | --- | --- | --- | --- | --- | --- |
| **UPDRS part III score** | **-0.196 (0.108)** | **-0.260 (0.006)** | **-0.184 (0.055)** | **-0.072 (0.453)** | **-0.185**  **(0.053)** | **-0.276 (0.169)** |
| **Total K-MMSE score** | **0.183 (0.056)** | **0.120 (0.211)** | **0.113 (0.241)** | **0.019 (0.843)** | **0.045**  **(0.637)** | **0.039 (0.688)** |

Values are presented as correlation coefficient (*P* value). Data are results of partial correlation analysis, adjusting for age, sex, disease duration, levodopa-equivalent dose increment per year, dopamine transporter availability in the posterior putamen, and intracranial volume.

**Supplementary Table S3.** Cox regression analysis of subcortical shape for the development of levodopa-induced dyskinesia

| Subcortical region | Side | Hazard ratio | 95% confidence interval | *P* value |
| --- | --- | --- | --- | --- |
| Caudate | Left | 0.79 | 0.46 – 1.36 | 0.397 |
|  | Right | 0.70 | 0.46 – 1.04 | 0.079 |
|  | Bilateral | 0.69 | 0.42 – 1.15 | 0.155 |
| Putamen | Left | 0.66 | 0.38 – 1.15 | 0.141 |
|  | Right | 0.75 | 0.54 – 1.02 | 0.068 |
|  | Bilateral | 0.66 | 0.43 – 1.03 | 0.069 |
| Pallidum | Left | 0.85 | 0.54 – 1.31 | 0.457 |
|  | Right | 0.75 | 0.49 – 1.13 | 0.169 |
|  | Bilateral | 0.75 | 0.46 – 1.22 | 0.244 |
| Thalamus | Left | 0.70 | 0.51 – 0.95 | 0.023 |
|  | Right | 0.64 | 0.46 – 0.90 | 0.009 |
|  | Bilateral | 0.62 | 0.43 – 0.89 | 0.010 |
| Hippocampus | Left | 0.85 | 0.58 – 1.25 | 0.402 |
|  | Right | 0.82 | 0.47 – 1.42 | 0.468 |
|  | Bilateral | 0.78 | 0.46 – 1.33 | 0.363 |
| Amygdala | Left | 0.69 | 0.44 – 1.08 | 0.105 |
|  | Right | 0.66 | 0.45 – 1.11 | 0.135 |
|  | Bilateral | 0.64 | 0.41 – 1.07 | 0.109 |

Data are results of Cox regression analysis, adjusting for age, sex, disease duration, levodopa-equivalent dose increment per year, dopamine transporter availability in the posterior putamen, and intracranial volume.

**Supplementary Figure S1.** Uncorrected *P*-map of the regional subcortical shape between the PD-rLID and PD-vLID.

**
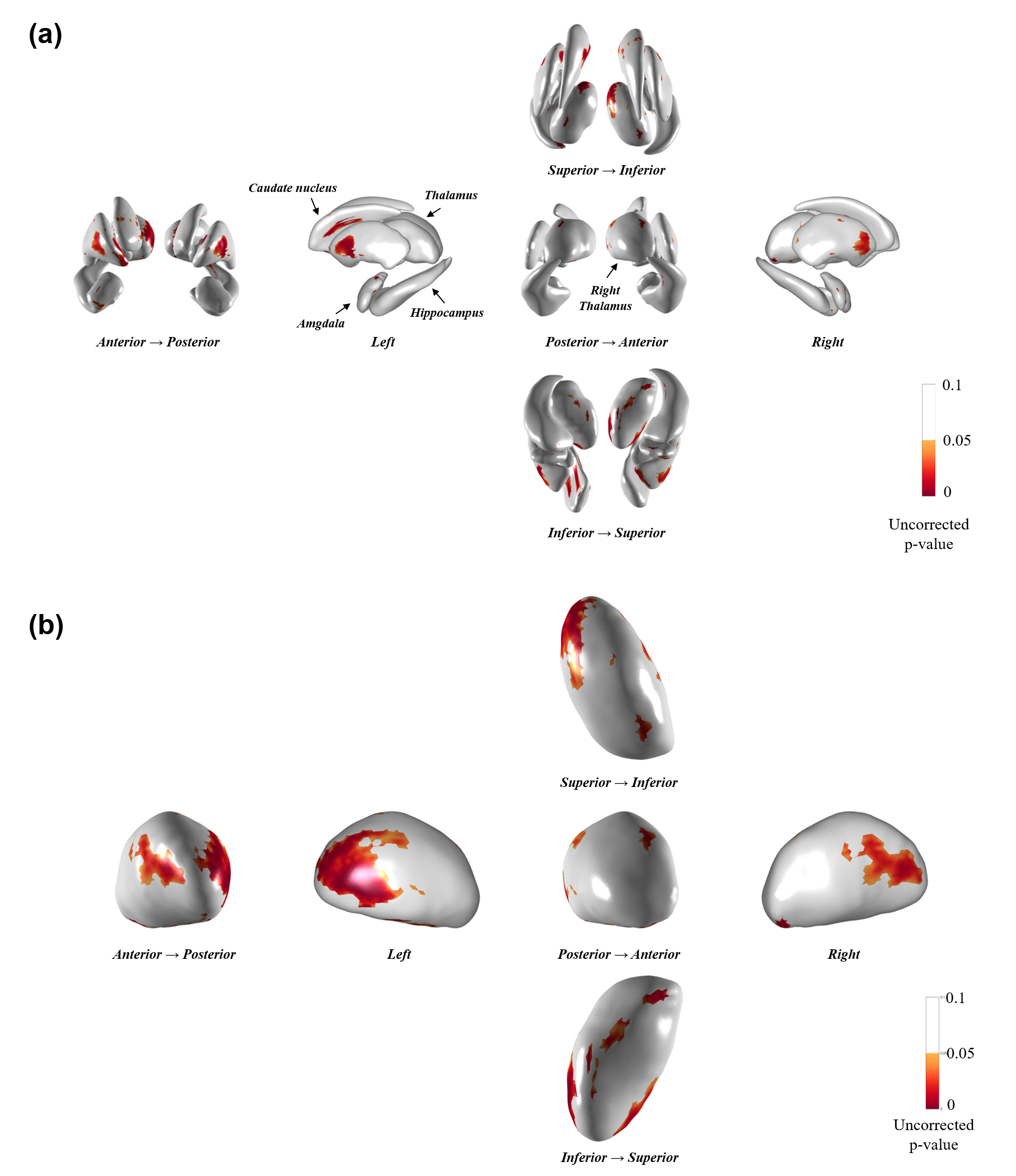
**

The results are based on an analysis of covariance, after adjusting for age, sex, disease duration, levodopa-equivalent dose increments per year, dopamine transporter availability in the posterior putamen, and intracranial volume. The uncorrected *P*-map indicates the subcortical regions that showed inward deformation in the latter group at the vertex level (yellow to red color). Abbreviations: PD-rLID, PD group resistant to levodopa-induced dyskinesia; PD-vLID, PD group resistant to levodopa-induced dyskinesia.
